# A Comparative Assessment of Acceptance of Different Types of Functional Appliances

**DOI:** 10.7759/cureus.48862

**Published:** 2023-11-15

**Authors:** Anju Jha, Richa Shree, Sovendu Jha, Goldi Sinha, Zainab Hassan, Kajol Kumari

**Affiliations:** 1 Department of Pediatric and Preventive Dentistry, Patna Dental College and Hospital, Patna, IND; 2 Department of Orthodontics and Dentofacial Orthopaedics, Buddha Institute of Dental Sciences and Hospital, Patna, IND; 3 Department of Orthodontics and Dentofacial Orthopaedics, Vishalnath Hospital, Hazipur, IND; 4 Department of Orthodontics and Dentofacial Orthopaedics, Sanjeevani Dental Clinic, Patna, IND; 5 Department of Orthodontics and Dentofacial Orthopaedics, New Apollo Oral and Dental Care Center, Hajipur, IND

**Keywords:** fabrication, pronunciation, malocclusions, acceptance, functional appliance

## Abstract

Background

Modern clinical orthodontics' functional appliances, a well-established modality of treatment, exhibit an amazing diversity of design. Clinical findings show that people have difficulty adjusting to these devices due to their size and unfixed positioning inside the mouth and that patient adaptation may vary based on the type of orthodontic functional appliance employed. Despite the fact that they appear to inflict more pain and soreness than, for example, removable plates, the effects of various orthodontic functional appliances on patients' acclimation have not yet been researched.

Aim

The current study's goal was to assess how different functional appliances' shapes and designs affected patients' willingness to accept them.

Materials and methods

About 20 adult volunteers (10 males and 10 females, age 18-32 years) with marked Class II division 1 malocclusion and not familiar with orthodontic appliances were selected as test subjects. Impressions for working casts were taken, and construction bites were prepared for the fabrication of eight functional appliances of various designs for each individual test subject. These appliances had eight design variations. There were three tests: one for speech effects, one for initial acceptance, and one for final acceptance after wearing different scales.

Results

Overall, the correlation between the quality of speech and pronunciation after wearing the appliance and the type of functional appliance was statistically significant. The quality of speech and pronunciation after wearing the appliance was maximum in frequency range 1 (FR1), while it was minimum in the medium-size activator. The difference was statistically significant (p=0.001). Overall, the correlation between the comfort and acceptability of functional appliances after wearing them and the type of functional appliance was statistically significant. The acceptance of functional appliances after wearing was maximum in FR1, while it was minimum in the medium-size activator. The difference was statistically significant (p=0.001). Overall, the correlation between the type of functional appliance and initial acceptance was significant statistically, with the maximum initial acceptance in medium-sized activators and the minimum initial acceptance in small bionators (p=0.001).

Conclusion

The study's findings show that patient acceptance of various kinds of functional appliances varies significantly.

## Introduction

The patient's collaboration has a significant impact on the treatment outcomes of portable orthodontic equipment, regardless of their specific therapeutic goal and mechanism of action. Discipline, patience, endurance, attitude towards therapy, and supervision by parents are just a few of the personality traits that influence patients receiving orthodontic therapy to utilize a removable device [1].

While a clinician can affect a patient's enthusiasm, the effectiveness of this effect is constrained because the patient's pertinent personality traits largely rely on his or her unique surroundings and learning and are therefore outside the scope of conversations with the clinician. Removable orthodontic appliances are known to occasionally result in uncomfortable tactile experiences, irritation of the mucosa, straining of delicate tissues, and tongue dislocation. Additionally, they may cause functional limitations in speech, deglutition, and breathing, as well as have aesthetic effects. These appliance wear side effects serve as negative cues, interfering with the process of adaptation and lowering the acceptability of the appliance [2,3].

According to the findings of a new research study, the degree of primary discomfort and tenderness felt following the implantation of the initially placed orthodontic appliance may be used to predict whether orthodontic devices and therapy, in general, would be accepted. Therefore, it would seem that physicians may increase the acceptance of the appliance by choosing a design that would allow for pleasant wear and ease of adaptation to the appliance [4-7].

Functional appliances, which are an established mode of treatment in modern clinical orthodontics, show a remarkable diversity of design [5-9]. Considering the dimensions and unfixed placement inside the mouth, it's evident from clinical observations that individuals do not easily adjust to these devices and that patients' adaptability may differ depending on the kind of orthodontic functional appliance used. The impact of various forms of orthodontic functional appliances on patients' acclimation has not yet been studied, despite the fact that they appear to cause greater discomfort and tenderness than, for example, removable plates [10-14]. The current study's goal was to assess how different functional appliances' shapes and designs affected patients' willingness to accept them.

## Materials and methods

**Figure 1:**
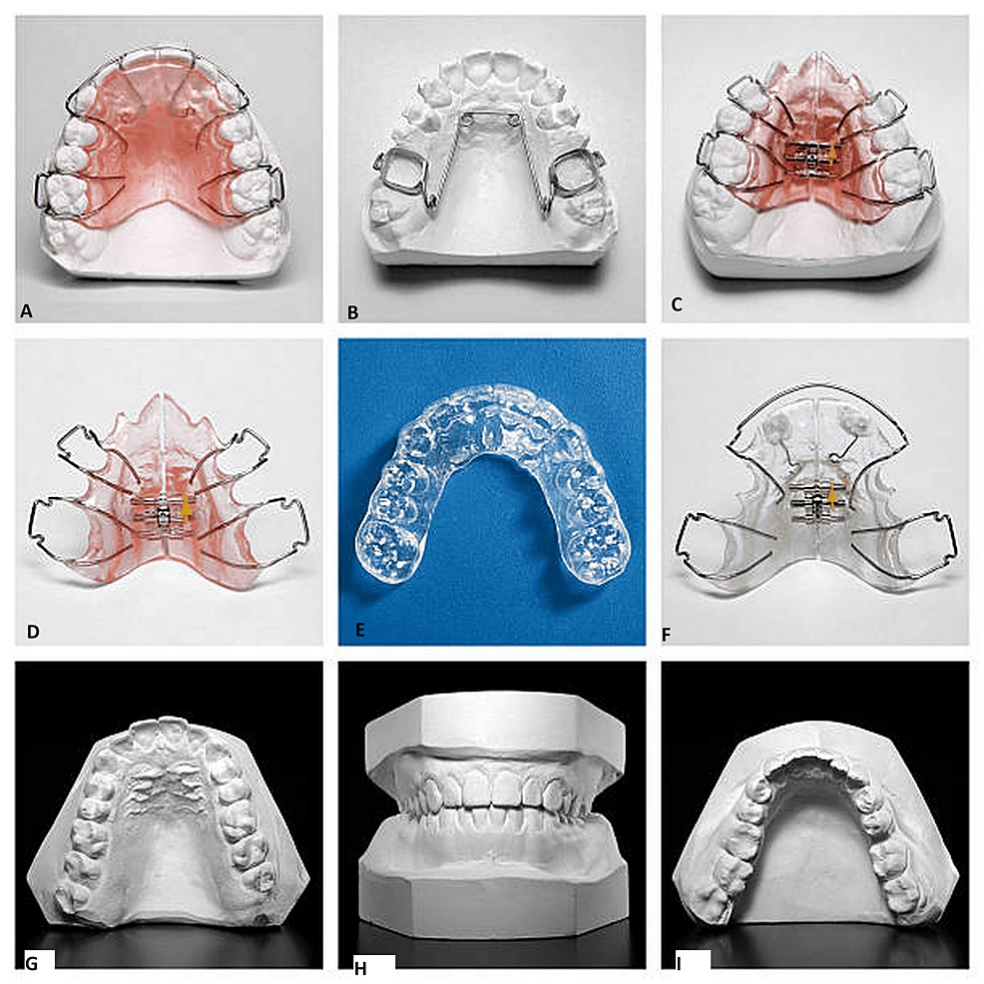
Functional appliances and the cast used for fabricating the appliances A: medium-size activator with extensive IO; B: large activator with moderate IO; C: small activator with moderate IO; D: functional corrector FR-I; E: medium-size elastic open activator with moderate IO; F: small horizontally split activator with flexible mandibular guidance and moderate IO; G, H, I: Cast of the patient used IO: Interocclusal distance

About 20 adult volunteers (10 males and 10 females, ages 18-32) with marked Class II division 1 malocclusion and not familiar with orthodontic appliances were selected as test subjects. Ethical approval was taken from the Ethical Committee of Patna Dental College and Hospital with institutional review board number IEC/PDCH/37. Impressions for working casts were taken, and construction bites were prepared for the fabrication of eight functional appliances of various designs for each individual test subject. A: These appliances had the following design variations: A: medium-size activator with extensive interocclusal distance (IO); B: large activator with moderate IO; C: small activator with moderate IO; D: functional corrector FR-I; E: medium-size elastic open activator with moderate IO; F: small horizontally split activator with flexible mandibular guidance and moderate IO; G, H, I: cast of the patient used (Figure 1).

All appliances had a resin base that was 2.0 mm thick overall. The distance between the maxillary first molar and mandibular first molars in the construction biting was used to calculate the IO. The quantity of IO varied between 2.0 and 5.0 mm for appliances with minimal IO, 5.0 to 7.5 mm for those with moderate IO, and 8.0 to 11.5 mm for those with substantial IO, depending on the personal occlusions of the study participants. The quantity of IO was kept consistent when various appliances of the same category were created for a certain subject.

The dimension of the appliance foundation, particularly the length of the lingual flanges, was associated with the size of the appliance, whether large, medium, or small.

In the bionator appliances, the lingual flanges extended 7.0 and 5.0 mm in the upward direction and 5.0 mm in the downward direction from the occlusal plane, respectively. It measured 8.0 and 6.0 mm in an upward and downward direction in the small-size activators, respectively. These readings were 11.0 and 7.0 mm for medium-sized appliances and 14.0 and 8.0 mm for large-sized activators, respectively, in both the upward and downward directions. Depending on the person's unique magnitude of sagittal discrepancy, the mandibular progression in the sagittal plane ranged from 2.0 to 6.0 mm, but it remained consistent for each study participant regardless of the kind of appliance.

Every functional appliance's volume was calculated by placing it in a measuring cylinder that was filled with water. Following are the results of a set of tests that were used to gauge the test participants' initial embrace of the aforementioned equipment.

Test 1: speech-related effects

The test volunteers were given a predetermined text in writing to read aloud, first with no appliance and then with eight appliances placed in their mouths sequentially and randomly. The subjects were given a one-minute break in between appliance changes, and following the insertion, they had 10 seconds to get used to the fresh appliance before turning their attention to the text.

A group of investigators, comprising a dental surgery assistant, an orthodontist, a theater educator, a speech-language therapist, and a school teacher, assessed the reading of the material after it had been recorded on tape. Eight more recordings from every participant were evaluated using the material spoken aloud without aid as a guide. On a five-point grading system, with one being the most accurate pronunciation, every examiner separately graded the study participant's pronunciation.

Test 2: initial acceptance

In this test, the individuals were asked to rate how comfortable each appliance felt when placed in the oral cavity based on their initial, unprompted impressions. In order to rule out any possible muddying of visual perception, the individuals were blindfolded. Each test subject was shown 56 pairings of the eight appliances. They were told to place the equipment in their mouths and to indicate each time which of the two they preferred to wear since it was more pleasant. In order to reduce potential weariness, the individuals were given brief rest intervals in between each session.

Test 3: acceptance after wearing

The test volunteers were directed to wear the devices for a total of 12 hours per day, one day each, in an arbitrary sequence that varied for every person. The individuals were then asked to rate each device's comfort on a six-point grading system, with one representing the most pleasant appliance.

Statistical analysis

For each test, the average ratings and preferred counts for each appliance were found, and Friedman's two-way ANOVA, which uses both the study participants and the appliance categories as sources of variation, was used to see if the differences between them were statistically significant. The Student-Neuman-Keuls all-pair-wise comparison approach was then used. The Friedman test as well as the Student-Neuman-Keuls test results were utilized to reject the null hypothesis with a p-value of 0.05.

## Results

The mean ratings given by investigators for the effect on speech and pronunciation in appliance A were 4.53± 0.34. Similarly, the ratings were 4.24± 0.67 in appliance B. The ratings for appliance C were 4.01± 0.23. There was no statistically significant difference in the results of these three appliances (p=0.891). The mean ratings given by investigators for the effect on speech and pronunciation in appliance D were 1.75±0.14. Similarly, the ratings were 3.82±0.35 in appliance E. The rating for appliance F was 3.67±0.46. The ratings in appliance G were 2.42±0.32. The ratings in appliance H were 2.63±0.42. The quality of speech and pronunciation was in the order of D>G>H>F>E>C>B>A.

The quality of speech and pronunciation after wearing the appliance was highest in appliances D, G, and H. The difference in findings in appliances D, G, and H was non-significant statistically (p=0.89). However, the difference in acceptance after wearing D, G, and H in comparison to the other five functional appliances was statistically significant (p<0.001). The quality of speech and pronunciation after wearing appliances C, B, and A was the lowest. The difference in values of acceptance after wearing appliances C, B, and A was not statistically significant (p=0.21). However, the difference in values of all other five appliances and C, B, and A was significant statistically (p<0.001). The values of F, E, and quality of speech and pronunciation after wearing the appliance were mediocre. The difference in values between appliances F and E was not statistically significant (p=0.89). However, the difference in values of all other five appliances and F and E was significant statistically (p<0.001).

Overall, the correlation between the quality of speech and pronunciation after wearing an appliance and the type of functional appliance was statistically significant. The quality of speech and pronunciation after wearing the appliance was maximum in frequency range 1 (FR1), while it was minimum in the medium-size activator. The difference was statistically significant (p=0.001) (Table 1).

**Table 1:** Mean ratings obtained in the test for speech effects SD: Standard deviation p<0.05 indicates statistical significance

Variables	Mean rating	SD	p-value
A	4.53	0.34	0.001
B	4.24	0.67	
C	4.01	0.23	
D	1.75	0.14	
E	3.82	0.35	
F	3.67	0.46	
G	2.42	0.32	
H	2.63	0.42	

The mean ratings given by investigators for the initial acceptance of appliance A were 1.20± 0.01. Similarly, the ratings were 2.92 ± 0.13 for appliance B. The ratings for appliance C were 4.23± 0.34. The mean ratings given by investigators for the effect on speech and pronunciation of appliance D were 11.57±1.26. Similarly, the ratings were 7.96 ± 0.98 in appliance E. The rating for appliance F was 6.23±0.87. The ratings in appliance G were 10.42±1.47. The ratings in appliance H were 11.81±1.25. The sequence of initial acceptance was in the order of A>B>C>F>E>G>D>H. The values of A, B, and C initial acceptance were the maximum. The difference in values of initial acceptance in appliances A, B, and C was not statistically significant (p=0.98). However, the difference in values of all other five appliances and A, B, and C was significant statistically (p<0.001). The values of initial acceptance were minimum in appliances G, D, and H. The difference in values of initial acceptance in appliances G, D, and H was not statistically significant (p=0.79). However, the difference in values of all other five appliances and G, D, and H was significant statistically (p<0.001). The values of F and E for initial acceptance were average. The difference in values of initial acceptance in appliances F and E was not statistically significant (p=0.67). However, the difference in values of all other five appliances and F and E was significant statistically (p<0.001). Overall, there was a statistically significant link between the type of functional appliance and how well it was accepted at first. The initial acceptance was best for medium-sized activators and worst for small bionators (p=0.001) (Table 2).

**Table 2:** Mean ratings for the second test of initial acceptance SD: Standard deviation p<0.05 indicates statistical significance

Variables	Mean rating	SD	p-value
A	1.20	0.01	0.001
B	2.92	0.13	
C	4.23	0.34	
D	11.57	1.26	
E	7.96	0.98	
F	6.23	0.87	
G	10.42	1.47	
H	11.81	1.25	

The mean ratings given by investigators for acceptance of the appliance after wearing it for appliance A were 4.67±0.24. Similarly, the ratings were 4.01± 0.21 for appliance B. The rating for appliance C was 3.82±0.18. The mean ratings given by investigators for the effect on speech and pronunciation for appliance D were 1.52±0.02. Similarly, the ratings were 3.21±0.14 in appliance E. The ratings in appliance F were 3.13±0.11. The ratings in appliance G were 1.75± 0.04. The ratings in appliance H were 2.21±0.03. The sequence of acceptance of the appliance after wearing was in the order of D>G>H>F>E>C>A>B.

The final acceptance of the appliance after wearing was minimal in appliances D, G, and H. The difference in findings in appliances D, G, and H was non-significant statistically (p=0.77). However, the difference in acceptance after wearing D, G, and H in comparison to the other five functional appliances was statistically significant (p<0.001). The values of acceptance after wearing were minimal in appliances C, A, and B. The difference in values of acceptance after wearing appliances C, A, and B was not statistically significant (p=1.21). However, the difference in values of all other five appliances and C, A, and B was significant statistically (p<0.001). The values of F and E for final acceptance after wear were mediocre. The difference in values between appliances F and E was not statistically significant (p=0.89). However, the difference in values of all other five appliances and F and E was significant statistically (p<0.001).

Overall, the correlation between the comfort and acceptability of functional appliances after wearing and the type of functional appliance was statistically significant. The acceptance of functional appliances after wearing was maximum in FR1, while it was minimum in medium-size activators. The difference was statistically significant (p=0.001) (Table 3).

**Table 3:** Mean ratings for response in the third test for final acceptance SD: Standard deviation p<0.05 indicates statistical significance

Variables	Mean rating	SD	p-value
A	4.67	0.24	0.002
B	4.01	0.21	
C	3.82	0.18	
D	1.52	0.02	
E	3.21	0.14	
F	3.13	0.11	
G	1.75	0.04	
H	2.21	0.03	

## Discussion

Modern clinical orthodontics' functional appliances, a well-established modality of treatment, exhibit an amazing diversity of design. Clinical findings show that people have difficulty adjusting to these devices due to their size and unfixed positioning inside the mouth and that patient adaptation may vary based on the type of orthodontic functional appliance employed. Despite the fact that they appear to inflict more pain and soreness than, for example, removable plates, the effects of various orthodontic functional appliances on patients' acclimation have not yet been researched [15-18]. The current study's goal was to assess how different functional appliances' shapes and designs affected patients' willingness to accept them.

In this study, overall, the correlation between the quality of speech and pronunciation after wearing an appliance and the type of functional appliance was significant statistically. The quality of speech and pronunciation after wearing the appliance was maximum in FR1, while it was minimum in the medium-size activator. The difference was statistically significant (p=0.001). The mean ratings given by investigators for the effect on speech and pronunciation in appliance A were 4.53± 0.34. Similarly, the ratings were 4.24± 0.67 in appliance B. The ratings for appliance C were 4.01± 0.23. There was no statistically significant difference in the results of these three appliances (p=0.891). The mean ratings given by investigators for the effect on speech and pronunciation in appliance D were 1.75±0.14. Similarly, the ratings were 3.82±0.35 in appliance E. The rating for appliance F was 3.67±0.46. The ratings in appliance G were 2.42±0.32. The ratings in appliance H were 2.63±0.42. The quality of speech and pronunciation was in the order of D>G>H>F>E>C>B>A.

The quality of speech and pronunciation after wearing the appliance was highest in appliances D, G, and H. The difference in findings in appliances D, G, and H was non-significant statistically (p=0.89). However, the difference in acceptance after wearing D, G, and H in comparison to the other five functional appliances was statistically significant (p<0.001). The quality of speech and pronunciation after wearing the appliance was minimal in appliances C, B, and A. The difference in values of acceptance after wearing appliances C, B, and A was not statistically significant (p=0.21). However, the difference in values of all other five appliances and C, B, and A was significant statistically (p<0.001). The values of F and E for quality of speech and pronunciation after wearing the appliance were mediocre. The difference in values between appliances F and E was not statistically significant (p=0.89). However, the difference in values of all other five appliances and F and E was significant statistically.

The research's findings are comparable to those of Sergl et al.'s study [1]. According to the results of a recent study, it may be possible to anticipate whether orthodontic devices and therapy, in general, would be accepted based on the level of first soreness and tenderness experienced after the implantation of the initially placed orthodontic appliance. It follows that doctors might improve the acceptance of the equipment by selecting a design that would make it comfortable to wear and make adaptation to the appliance simple.

It is well-recognized that removable orthodontic appliances can occasionally cause uncomfortable tactile sensations, mucosal irritation, sensitive tissue strain, and tongue dislocation. Additionally, they could impair breathing, deglutition, and speech, in addition to having aesthetic impacts. These side effects of appliance use act as warning signs, impeding the process of adaptation and diminishing the device's acceptance [19-22].

In this study, the correlation between the type of functional appliance and initial acceptance was significant statistically, with the maximum initial acceptance in medium-size activators and the minimum initial acceptance in small bionators (p=0.001). The sequence of initial acceptance was in the order of A>B>C>F>E>G>D>H. The values of A, B, and C initial acceptance were the maximum. The difference in values of initial acceptance in appliances A, B, and C was not statistically significant (p=0.98). However, the difference in values of all other five appliances and A, B, and C was significant statistically (p<0.001). The values of initial acceptance were minimum in appliances G, D, and H. The difference in values of initial acceptance in appliances G, D, and H was not statistically significant (p=0.79). However, the difference in values of all other five appliances and G, D, and H was significant statistically (p<0.001). The values of F and E for initial acceptance were average. The difference in values of initial acceptance in appliances F and E was not statistically significant (p=0.67). However, the difference in values of all other five appliances and F and E was significant statistically (p<0.001).

The findings were similar to the findings of the study. The research's findings are comparable to those of Sergl et al.'s study [1]. Regardless of the precise therapeutic goal and method of action of portable orthodontic equipment, the patient's cooperation has a substantial impact on the treatment outcomes. Just a few of the personality factors that affect patients undergoing orthodontic therapy's use of a detachable device include discipline, patience, endurance, attitude toward therapy, and parental monitoring. Although a clinician can influence a patient's excitement, the effectiveness of this impact is limited because the patient's relevant personality qualities mostly depend on his or her particular upbringing and education and are thus outside the purview of dialogues with the therapist [15-18,23-24].

In this study, the sequence of acceptance of the appliance after wearing was in the order of D>G>H>F>E>C>A>B. Overall, the correlation between the comfort and acceptability of functional appliances after wearing and the type of functional appliance was statistically significant. The acceptance of functional appliances after wearing was maximum in FR1, while it was minimum in medium-size activators. The difference was statistically significant (p=0.001).

The mean ratings given by investigators for acceptance of the appliance after wearing it for appliance A were 4.67±0.24. Similarly, the ratings were 4.01± 0.21 for appliance B. The rating for appliance C was 3.82±0.18. The mean ratings given by investigators for the effect on speech and pronunciation for appliance D were 1.52±0.02. Similarly, the ratings were 3.21±0.14 in appliance E. The ratings in appliance F were 3.13±0.11. The ratings in appliance G were 1.75± 0.04. The ratings in appliance H were 2.21±0.03.

The final acceptance of the appliance after wearing was minimal in appliances D, G, and H. The difference in findings in appliances D, G, and H was non-significant statistically (p=0.77). However, the difference in acceptance after wearing D, G, and H in comparison to the other five functional appliances was statistically significant (p<0.001). The values of acceptance after wearing were minimal in appliances C, A, and B. The difference in values of acceptance after wearing appliances C, A, and B was not statistically significant (p=1.21). However, the difference in values of all other five appliances and C, A, and B was significant statistically (p<0.001). The values of F and E for final acceptance after wearing were mediocre. The difference in values between appliances F and E was not statistically significant (p=0.89). However, the difference in values of all other five appliances and F and E was significant statistically (p<0.001).

However, the findings of this study indicate that variables determining the primary acceptance of an appliance should always be taken into account, particularly since the level of discomfort experienced during therapy may have a significant impact on a patient's approval of the therapy throughout its duration and subsequent compliance. A successful course of treatment is greatly influenced by a patient's ability to consistently wear an appliance; therefore, it stands to reason that more agreeable equipment would be worn by the individual in question more readily [20-25].

Limitations of the study include a relatively small sample size of only 20 adult volunteers with a specific malocclusion type. This limited sample might not fully represent the broader population seeking orthodontic treatment, potentially affecting the generalizability of the findings. Other types of malocclusions were not considered, which restricts the study's applicability to a broader range of orthodontic cases. The study evaluated the acceptance and comfort of functional appliances immediately after wearing them. Long-term effects, including adjustments in patients' willingness to accept the appliances over extended usage, were not explored. The assessment of patient acceptance was based on self-reported scales, which are subjective measures. Objective measures, such as physiological responses or behavioral observations, could provide a more comprehensive understanding.

## Conclusions

The study's outcomes underscore the importance of considering the design and type of orthodontic functional appliances when addressing patient adaptation and acceptance. The findings indicate that certain appliance designs, such as the functional corrector FR1, may offer better speech quality, comfort, and overall acceptability compared to others, like the medium-size activator or small bionator. These insights can inform orthodontic treatment decisions and contribute to improving the patient experience during orthodontic care.
